# The Educational Influence of Sanatorium Treatment

**Published:** 1909-06-19

**Authors:** J. E. Bullock


### THE GENERAL PRACTITIONER'S COLUMN.

[Contributions to this Column are invited, and if accepted will be paid for.]

### THE EDUCATIONAL INFLUENCE OF SANATORIUM TREATMENT.

By J. E. BULLOCK, M.D.

The educational influence of a stay in a sanatorium is enormous. I am confident that, as more and more patients, when they exchange sanatorium life for their usual avocations at home or for another sphere of work altogether, continue to carry out the principles and practices learnt during their stay, so more and more will consumption be prevented. Persons while in a sanatorium learn not only how to arrest the disease in themselves, but how to detect the possibilities of its existence in others; and not only so, but to warn them against that manner of living which they now know to be injurious.

Life in a sanatorium must be an astounding experience to all who enter such an institution for the first time. Although they may have read a good deal on the subject and think they know what to expect, there are many things which they have not up to then fully believed in. An early lesson learnt is that, provided the body is kept quite warm by suitable covering, it matters nothing how cold the respired air is; also that the injurious effect of "damp" depends on the chilling effect it has on the body, and that exposure to a storm of rain is harmless if the body is not allowed to be chilled by damp clothes. Life in a sanatorium is practically life in the open air; the windows are freely open day and night, so as to approximate the conditions to the outside air as much as possible. There is found to be no more "draught" than there is out of doors; the wind may be felt to be blowing from a certain direction, as it always must more or less, but it is recognised that that is not a draught any more than a similar experience out of doors; the remedy applied is the closure of the windows and doors open to the direct current of the wind, if it is too chilling to the surface of the body, and to admit the air from a direction opposite to that of the prevailing wind.

The walls of a sanatorium give stability to the structure, form a protection against excessively bad weather, and enclose the rooms and offices necessary to any dwelling-house. When this is provided for, the rest of the building is entirely open to the air. Twenty-four hours in a sanatorium teaches patients that they can be freely exposed to the air in a manner which they never dreamed of in their own homes, and when once they have learnt this they will not be content to live in the stuffy rooms to which they have been previously accustomed.

In a sanatorium careless expectoration is never seen; those who have any expectoration invariably carry sputum flasks, and use them as required. Smokers also use sputum flasks for ordinary salivation. Seeing that the great means of spread of tuberculosis is by sputum carelessly ejected on to the ground and allowed to dry and disseminate, the lesson learnt, *never* to expectorate except into a sputum flask, must be of enormous advantage to the community. The general public are being taught *not to spit*, but many disregard this, either from ignorance, indifference, or wilful neglect. The objection which some consumptives and many non-consumptives urge against carrying a sputum-flask is that it would look "strange to others," but it is learnt that the flask need not appear to others at all. With very little practice it can be so used that no one notices it, the flask being readily concealed in the hand or handkerchief, and the sputum noiselessly ejected. Each patient is so impressed with the danger of swallowing sputum, whereby the tubercle bacilli may reach the intestines, that thereafter he will always use his flask, and can generally do so without attracting attention. If he should be compelled to use his handkerchief to receive the sputum, he has been taught to steep the handkerchief at the earliest opportunity in some disinfecting fluid and afterwards to have it boiled. He has learnt that his sputum-flask must be carefully cleansed and disinfected, and that forks, spoons, and cups which have touched his lips must be boiled.

In the matter of diet a most useful education is obtained in a sanatorium. Many consumptives do not know the essentials of food and how to regulate their diet. It is generally taught that they should take a good deal of fatty food. Milk enters largely into the dietary: it may be drunk instead of water or beer at lunch and dinner, in the early morning before breakfast, in the course of the forenoon, and at bedtime; as much as possible of other food is taken, with a good proportion of meat. A sanatorium may take two classes of patients: (1) those in an early stage who are fairly robust, (2) those in whom the disease is advanced and perhaps advancing. Such cases are dieted on different lines. The former are not often suffering from dyspepsia, and they do well on a full diet; the latter are usually more or less dyspeptic and require careful dieting. They are instructed in a proper dietary in a sanatorium.

Again, patients learn to test their condition by a careful observation of their temperature. Each patient has a thermometer which he learns to use properly. A reliable temperature can best be obtained in the rectum. This method is rarely practised by those who have not been instructed in its necessity. The more convenient method by the mouth often registers a degree below the proper temperature. Over-exercise or over-excitement, as factors which raise the temperature, are carefully guarded against in a sanatorium. Though no patient should be repeatedly studying his temperature, the lessons which he learns about its indications can be carefully borne in mind when he returns to his usual mode of life. He has learnt what exercise he should take and what work he can accomplish without undue elevation of temperature. Many young men come to a sanatorium direct from an unsuitable life in towns and in close offices. The opportunities indicated to them of changing their mode of life in the future are of great advantage. For many it is desirable that they should take up another calling altogether and seek some outdoor occupation, if they are at all suited to it. In a large sanatorium they can gain practice and experience in gardening, the management of poultry and cattle, agriculture, land-surveying, and such like. Since many young men wish to go to the Colonies for the sake of the outdoor life, they can judge during their stay at a sanatorium whether they are at all fitted for it; and, if so, they may try, under medical guidance, the outdoor occupation which seems best suited to their tastes and capabilities. If a patient on leaving a sanatorium is free to choose his place of residence, he has learnt that the best site is a gentle slope towards the south, on a porous soil, with, if possible, rising ground to the back; that his rooms should have a southerly aspect; and that he must provide for a free current of air through his rooms by means of windows (which can be left constantly open) and open doors as required.

As a rule the consumptive patient readily adapts himself to sanatorium life; he is practically never despondent even when everyone else sees that his condition is hopeless. There are, however, a few exceptional cases in which discontent prevails: the patient is never satisfied with the advice given him. His constant complaint is, "I shall never get better under this kind of treatment." He has enough hopefulness to lead him to talk about getting better, but he has not enough strength of will and purpose to enable him to do so. At length he feels himself going down hill and he gives up the struggle at once. Such a patient derives no benefit from a sanatorium. It may be thought that constant association with others affected with the same disease might tend to encourage a morbid self-inspection, but my experience is that though patients will always discuss their symptoms among themselves, they rarely, if ever, take a depressed view of them. They encourage each other, and any throw-back which a convalescent may meet with acts as a warning to those who are tempted to do too much, and some knowledge of the acute condition in others may deter slight cases from being reckless.

In some instances smoking may be indulged in too freely. Here the patient must make a rule not to smoke until after the mid-day meal, never immediately before the evening meal, and not at all indoors.

